# The Bifidogenic Effect Revisited—Ecology and Health Perspectives of Bifidobacterial Colonization in Early Life

**DOI:** 10.3390/microorganisms8121855

**Published:** 2020-11-25

**Authors:** Himanshu Kumar, Maria Carmen Collado, Harm Wopereis, Seppo Salminen, Jan Knol, Guus Roeselers

**Affiliations:** 1 Danone Nutricia Research, 3584 CT Utrecht, The Netherlands; Himanshu.kumar@danone.com (H.K.); harm.wopereis@danone.com (H.W.); jan.knol@wur.nl (J.K.); 2 Department of Biotechnology, Institute of Agrochemistry and Food Technology-Spanish National Research Council (IATA-CSIC), Paterna, 46980 Valencia, Spain; mcolam@iata.csic.es; 3 Functional Foods Forum, Faculty of Medicine, University of Turku, 20500 Turku, Finland; seppo.salminen@utu.fi; 4 Laboratory for Microbiology, Wageningen University, 6708 PB Wageningen, The Netherlands

**Keywords:** microbiome, symbiosis, co-evolution, caesarean section, milk, host derived glycans, human mik oligosaccharides, probiotics, prebiotics, synbiotics

## Abstract

Extensive microbial colonization of the infant gastrointestinal tract starts after parturition. There are several parallel mechanisms by which early life microbiome acquisition may proceed, including early exposure to maternal vaginal and fecal microbiota, transmission of skin associated microbes, and ingestion of microorganisms present in breast milk. The crucial role of vertical transmission from the maternal microbial reservoir during vaginal delivery is supported by the shared microbial strains observed among mothers and their babies and the distinctly different gut microbiome composition of caesarean-section born infants. The healthy infant colon is often dominated by members of the keystone genus *Bifidobacterium* that have evolved complex genetic pathways to metabolize different glycans present in human milk. In exchange for these host-derived nutrients, bifidobacteria’s saccharolytic activity results in an anaerobic and acidic gut environment that is protective against enteropathogenic infection. Interference with early-life microbiota acquisition and development could result in adverse health outcomes. Compromised microbiota development, often characterized by decreased abundance of *Bifidobacterium* species has been reported in infants delivered prematurely, delivered by caesarean section, early life antibiotic exposure and in the case of early life allergies. Various microbiome modulation strategies such as probiotic, prebiotics, synbiotics and postbiotics have been developed that are able to generate a bifidogenic shift and help to restore the microbiota development. This review explores the evolutionary ecology of early-life type *Bifidobacterium* strains and their symbiotic relationship with humans and discusses examples of compromised microbiota development in which stimulating the abundance and activity of *Bifidobacterium* has demonstrated beneficial associations with health.

## 1. Introduction

Intestinal microbiota development in early life is very dynamic and is in synergy with anatomical, intestinal physiology, immune and neurological development [1]. Deviations and misconfigurations in its structure and function may contribute to pathologies and chronic diseased states [2]. Timing of the first microbial exposure to the developing embryo is often debated [3]: some reports suggest that microbial exposure and colonization already happens “*in utero*” [4,5,6] while others have pointed out that more detailed studies are required to verify this observation [3]. 

Undoubtedly, there are many more parsimonious routes by which microbial inoculation may proceed, including early exposure to vaginal and fecal microbiota at birth, ingestion of epidermal skin microbes and viable microbes present in breast milk, as well as the inherently close interactions between the neonate and mother [7]. The subsequent colonization process is a balance between influx of microbes and niche adaptability [8].

These first microbial pioneers become key players in the assembly of a complex ecosystem that follows distinct successional stages with potential long term health consequences [9]. The formation of this complex ecosystem is influenced by multiple factors including host genetics, mother’s microbiota, gestational age, medical practices, mode of delivery, diet, life style, familial environment, presence of pets, infectious diseases and antimicrobial therapies [10]. Although microbiota assembly in the infant gut is not strictly deterministic, there is an overarching directionality of microbial succession strongly driven by early life nutrition, specifically human breast milk. 

Human milk not only provides optimal nutrition for the infants but is also a reservoir of microbes mainly within the genera *Staphylococcus* and *Streptococcus* but also *Bifidobacterium*, *Propionibacteria*, *Pseudomonas*, *Bacteroides* and *Parabacteroides* [11,12]. *Bifidobacterium* is the most abundant genus in the breastfed infant’s gut and is considered a true “keystone” taxon with a strong eco-physiological impact on microbiota composition and activity. Therefore, *Bifidobacterium* spp. may serve as a marker of healthy microbiota development and breast-feeding practices. The purpose of this review is to highlight the central role of bifidobacteria as keystone organisms in early life, and compare their distinct ecophysiology with other members of the early-life gut microbiota [8]. Furthermore, we aim to provide mechanistic insights which support the application of bifidobacteria as microbiome modulators (in conjunction with prebiotics) to restore compromised microbiota development linked to mode of delivery, antibiotic exposure, prematurity and childhood pathology such as allergy. 

## 2. Ecological Drivers of Acquisition and Succession of Bifidobacteria

### 2.1. Mode of Delivery, Antibiotics and Diet

Immediately after birth, the infant gut is still rich in oxygen and offers a favorable habitat for facultative anaerobic microorganisms such as *Staphylococcus* spp., *Streptococcus* spp., *Enterobacter* spp. and other members of family Enterobacteriaceae [10]. These pioneering species play an important role in the rapid transition from a microbiome dominated by taxa that tolerate or thrive under limited oxygen to microbiome dominated by strictly anaerobic taxa such as *Clostridium*, *Bacteroides*, *Eubacterium* and *Bifidobacterium* spp. [2,10]. However, birth via caesarean section (c-section) interrupts this program of microbiota acquisition and colonization since there is no contact with the maternal vaginal and fecal microbiota and the perineal skin. In addition, c-section born infants are often exposed to maternal prophylactic antibiotic administration and c-section birth has been shown to adversely affect breastfeeding initiation, milk supply and infant breastfeeding receptivity compared to vaginal deliveries, which may further compromise early life microbiota development. [13]. 

Instead, the guts of c-section-delivered infants are typically first colonized by human skin and oral cavity associated bacteria, which include *Staphylococcus* spp., *Streptococcus* spp., *Veillonella* spp., *Propionibacterium* spp., *Corynebacterium* spp. and *Acinetobacter* spp. [7,14,15]. Recently, a large study with 596 infants confirmed microbiota differences observed in c-section born infants [16]. Notably, this study reported that in vaginal born infants, commensal genera, such as *Bacteroides* and *Bifidobacterium* (such as *Bifidobacterium longum* and *Bifidobacterium breve* and *Bifidobacterium infantis*)*,* made up 68% of the total genus richness, while c-section born infants were depleted of these commensal genera and instead were enriched by species within the genera *Enterococcus, Staphylococcus, Streptococcus, Klebsiella, Enterobacter* and *Clostridium*, all of which are more characteristic for hospital environments and hospitalized preterm babies [16]. In addition, c-section delivery is often accompanied with varying use of medications, including prophylactic antibiotics, which further adds to the disrupted transfer of maternal gut microbiota. 

It has been shown that direct administration of antibiotics to neonates or indirect through feto-placental circulation has pervasive effects on gut microbiota composition, and is associated with adverse immune outcomes such as allergies and atopies [17], and metabolic health outcomes such as obesity [18]. Fouhy et al. demonstrated that antibiotic administration to term neonates led to a relative increase in fecal Proteobacteria and a decrease in Actinobacteria, particularly *Bifidobacterium* spp., representing deviation from normal microbiota development [19]. Intriguingly, it was also shown that intrapartum antibiotic administration not only led to differences in infant gut microbiota but also affected breast milk microbiota composition [20]. 

Upon delivery, breast milk is the most significant factor which impacts maturation of the gut microbiota. It has been shown that formula-fed infants exhibit a more diverse microbiota than breastfed infants [9,21,22]. The microbiota of vaginally delivered breast-fed infants is dominated by the Phylum Actinobacteria, while formula-fed infants adopt a more diverse microbiota [23]. Moreover, the cessation of breast feeding is associated with a steep reduction in the levels of bifidobacteria and an accelerated increase in members of Firmicutes and Bacteroides, which further substantiates the key role of the milk–bifidobacteria interaction in shaping the human gut microbiota [9].

### 2.2. Establishment of Bifidobacterium: A Keystone Genus

Bifidobacteria are regarded as a “keystone” taxon in the early life gut microbiota. The keystone organism concept is a fundamental principle in theoretical and applied ecology. The term was first introduced in a study on a rocky intertidal ecosystem in California. When the top predator (a starfish) was removed, the community collapsed, prompting the architectural analogy with the keystone of an arch [24]. Keystone organisms are important drivers of community structure and integrity, and their influence is non-redundant. Their activity and biotic interactions disproportionately determine species assemblages and the rates of material and energy flow across entire communities. These taxa have a unique and crucial role in microbial communities, and their removal can cause a dramatic shift in microbiome structure and functioning.

By their broad impact on microbiota composition function, keystone members are also likely to exert strong direct and indirect effects on host physiology and may be essential for host homeostasis and health [25]. In early life gut microbiota, *Bifidobacterium* and *Bacteroides* drive microbiota development by maintaining a strict anaerobic environment, by producing and cross-feeding on metabolites such as short chain fatty acids (SCFA). SCFA result in a low pH, which is among the main mechanisms of ecological resistance against pathogens [26,27]. The failure of these keystone taxa to colonize and drive succession may lead or contribute to the development of chronic diseases [28]. For instance, antibiotics administration has been clearly demonstrated to impair proliferation of these keystone taxa, which could in turn disturb microbial interaction with the immune system, particularly during critical stages of development. This failure of cross talk between keystone microbes and immune cells is thought to be an important factor in the development of allergies, metabolic disorders and infectious diseases [29].

Physiologically, the keystone function of bifidobacteria is strongly linked to their unique metabolic capacity and genomic architecture. Bifidobacteria are genetically adapted to utilize specific glycans of human milk, thus representing an intriguing example of host-microbe coevolution into mutualistic symbiosis.

## 3. Evolutionary and Eco-Physiological Attributes of a Bifidogenic Milieu

### 3.1. Evolutionary Ecology

The heterofermentative genus *Bifidobacterium* takes its name from its characteristic Y-shaped cells (in Latin, bifidus means cleft or divided into two parts). Isolation of bifidobacteria from infant feces provided first indications of their ecological relevance in the human gut [30]. Interestingly, bifidobacteria were also isolated from the gut of multicellular social organisms such as birds, mammals and social insects, which implies that bifidobacteria could be transmitted vertically [31,32]. In the gut, growth of bifidobacteria is nurtured by glycans through feeding or cross-feeding activity [33]. The unique genetic make-up of *Bifidobacterium* species gives an advantage to outcompete other gut commensals in metabolizing glycans present in human milk [30]. These two exceptional features—maternal transfer and genetic constitution—distinguish bifidobacteria from all other commensal gut bacteria such as *Lactobacillus species.*

**Figure 1:**
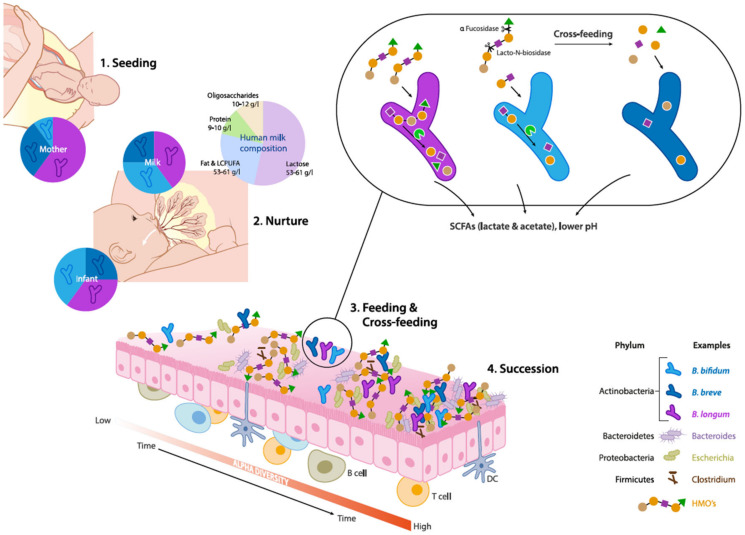
Schematic overview of eco-physiological factors driving maternal transmission, colonization and succession of *Bifidobacterium* species in the early life gut microbiota. (1) Seeding: Bifidobacteria are transmitted from mother to child during vaginal delivery. (2) Nurture: Human milk contains viable microorganisms, including Bifidobacterium species, which contribute to seeding the infant’s gut microbiota. Human milk oligosaccharides (HMOs) are minimally digested by the infant and metabolized by infant-type bifidobacteria by convergent mechanisms. (3) Feeding & Cross-feeding: different *Bifidobacterium* species and strains have distinct HMO degrading abilities resulting in varied HMO consumption behaviors. Degradation of HMO occurs sequentially with the removal monosaccharides and requires a multitude of enzymes with various glycosidic specificities. *Bifidobacterial* cross-feeding permits the sharing of resources to maximize nutrient consumption from the diet and highlights the cooperative nature of bifidobacterial strains and their role as ”key stone” species in the infant gut microbiota. (4) Succession: The combined activity of *Bifidobacterium* species contributes to the establishment and maintenance of a strict anaerobic environment and low pH by producing metabolites such as lactate and acetate. These conditions allow the successive establishment of butyrate producing taxa such as *Eubacterium* and *Anaerostipes spp* which are characteristic for a more matured microbiota.

Several studies investigating specific maternal–neonatal microbial transference have shown that mother and child often share genomically identical bifidobacterial strains belonging to *B. breve* and *B. longum* subsp. *longum, B. longum* subsp. *Infantis*, further substantiating vertical transmission of bifidobacteria (Figure 1) [34,35,36,37]. These findings provide initial insights as to why vaginal delivery provides a higher abundance of *Bifidobacterium* species in infants, over a c-section delivery.

In vaginally delivered breast-fed infants, the relative abundance of bifidobacteria could be over 90% but typically decreases to less than 5% in adults [38,39]. More specifically, breast-fed infants are predominated by the presence of *B. breve* spp., *B. bifidum, B. longum* subsp. *infantis* and *B. longum* subsp. *longum,* also known as infant type Human Resident Bifidobacteria (HRB) (Figure 1) [32]. On the other hand, adults are characterized by the presence of *B. adolescentis* and *B. catenulatum, B. pseudocatenulatum* and *B. longum* subsp. *longum* which are often termed as adult-type HRB [32,33]. Notably, *B. longum* subsp. *longum* was found to be predominant in both infant and adult gut. Hence, there is no strict distinction between “infant” type and “adult” type bifidobacteria, as some of the adult bifidobacteria such as *B. adolescentis* were shown to be vertically transferred to infants [40].

### 3.2. Genomic Features

The typical characteristics of bifidobacteria include their obligate anaerobism, peptidoglycan rich cell walls and heterofermentative metabolism. At present, there are 51 species and 10 subspecies of bifidobacteria reported, out of which 48 (sub)species have at least one genome sequenced (NCBI database) [41]. Pan-genome analyses of the *Bifidobacterium* genus revealed that 13.7% of the identified bifidobacterial genes are involved in carbohydrate metabolism, which is much higher than the other analyzed gut commensals [30,42]. Importantly, the core genome also encodes enzymes involved in the “bifid shunt”, which equips bifidobacteria with a unique evolutionary advantage of generating more ATP (per mole of glucose) in comparison to microorganisms using other carbohydrate fermentative pathways such as glycolysis [30]. Specifically, a Cluster of Orthologous Groups (COGs) representing α-amylases, ATP-binding cassettes (ABC) and phosphoenolpyruvate-phosphotransferase systems (PEP-PTS) were identified to be acquired during the course of evolution and give bifidobacteria a selective advantage in the highly competitive ecological niche of the early life gut [42]. Furthermore, comparative genome analyses of bifidobacteria, particularly *B. longum* subsp. *infantis* ATCC15697 and *B. bifidum* PRL2010, have revealed that these species are able to utilize a broad range of host-derived glycans (HMOs and mucin), further corroborating the genomic plasticity of *Bifidobacterium* spp. [30]. 

### 3.3. HMO Utilization

Human milk constitutes around 10–12 gm/L of oligosaccharides, which constitute the third most abundant component in milk [43,44] (Figure 1). HMOs are complex and structurally highly diverse, with over 200 different molecules that vary in size, Degree of Polymerization (DP), charge and sequence. The size distribution of HMOs ranges from 90% short chain oligosaccharides to 10% long chain oligosaccharides [45]. The most basic HMO structures are monomers of glucose (Glc), galactose (Gal) and *N*-acetylglucosamine (GlcNAc), and also contain fucose (Fuc) and/or *N*-acetylneuraminic acid (NeuAc) linked via several glycosidic bonds [46]. Most HMOs cannot be utilized by host digestive enzymes but are effectively utilized by gut microbiota. Therefore, HMOs play a pivotal role in shaping the infant gut microbiota, and actively promote beneficial bacteria, which is also termed as a “prebiotic” effect [47].

The abundance and prevalence of Bifidobacteria in the neonatal gut is attributed to their unique ability to catabolize HMOs [48]. For example, *B. longum* subsp. *infantis* and *B. breve* use specific ATP-binding cassette (ABC) transporters for internalization of intact oligosaccharides. Intracellular glycosyl hydrolases (GH) such as fucosidases, hexosaminidases and sialidases can further deconstruct the oligosaccharides [49,50] (Figure 1). Species such as *B. bifidum* have different HMO consumption capabilities. These taxa break down HMO via extracellular glycosidases into mono- and disaccharides, which are subsequently transported into the cells via permeases. Residues of this extracellular degradation allow cross-feeding of other types of bacteria including other *Bifidobacterium* species [51].

Preclinical experiments in conventional mice receiving a combination *B. bifidum* PRL2010, *B. longum* subsp. *infantis* ATCC15697, *B. adolescentis* 22L, and *B. breve 12L,* demonstrated a synergistic effect by acting directly or by cross-feeding on host or plant derived carbohydrates, which further led to the enrichment of murine gut glycobiome [52]. In contrast, other gut commensals such as *Lactobacillus* sp. and *Bacteroides* sp. show poor or limited capacity to utilize HMOs, respectively [50,53]. 

Short chain galacto-oligosaccharides (scGOS) and long chain fructo-oligosaccharides (lcFOS) in a ratio of 9:1 mimic the size distribution of HMOs and resembles functionality of breast milk [54]. Based on these observations, scGOS/lcFOS, and synthetic HMOs such as 2′Fucosyl Lactose (2′FL) and Lacto-N-neotetraose (LNnT), are being incorporated in infant formula. Altogether, these chemical constituents or prebiotics are aimed to increase bifidobacteria counts and thereby exert immune benefits. In clinical studies, it was demonstrated that 2′FL and LNnT modulated the gut microbiota exhibiting increased levels of Actinobacteria, specifically *Bifidobacterium* spp., and decreased levels of Firmicutes and Proteobacteria [55,56]. 

### 3.4. Effect on pH and SCFA Production

In breast-fed infants, HMO metabolism by gut microbiota is often associated with distinct SCFA profiles when compared to formula-fed infants, which are subsequently reflected in reduced fecal pH [57,58]. The prevalent SCFAs include acetate, butyrate, valerate, propionate and, to a lesser extent, branched chain fatty acids such as iso-butyrate and iso-valerate. Exclusively breast-fed infants are characterized by a higher relative proportion of acetate relative to other SCFAs in the gut, which was found to be independent of birth mode, sex, intrapartum antibiotics, site of recruitment and maternal body mass index [57,58,59]. Importantly, *Bifidobacteriaceae* was the only family which was significantly associated with fecal pH, although there are microbes (such as *Bacteroidaceae*) which can also utilize HMO [27,60]. In particular, bifidobacteria have evolved with specific mechanisms to produce acetic and lactic acids (in a molar ratio of 3:2) by utilization of glycans through the bifid shunt pathway (Figure 1). [61]. Based on these observations, fecal acetate is also regarded as a biomarker for bifidogenic activity and overall microbiota health in early life.

Physiologically, SCFAs have also been associated with both systemic effects such as immune modulation and local effects such as acting as an energy source for colonocytes. SCFAs may also provide colonization resistance against pathogens such as *Escherichia coli* O157:H7 [57,62]. In a clinical study, reduced fecal acetate at 3 months of age was associated with atopic wheeze observed 9 months later [63]. In addition, it was recently shown that prebiotic supplementation (short chain galacto-oligosaccharides (scGOS) and long-chain fructo-oligosaccharides (lcFOS)) in a partially hydrolyzed protein formula, led to increased *Bifidobacterium*/*Lachnospiraceae* ratio, which in turn was reflected in organic acid profiles with high acetate and lactate levels and low butyrate, propionate and branched chain SCFAs [64]. 

On the other hand, butyrate is considered as a marker for a healthy maturation of the gut microbiota when the infant diet diversifies with the introduction of solid foods [58,65]. Interestingly, acetate and lactate are important “cross-feeding” substrates for butyrate-producing bacteria such as *Faecalibacterium prausnitzii, Roseburia, Anaerostipes* spp. and *Eubacterium halli* (Figure 1) [66]. This gradual transition from a bifidogenic and acetogenic milieu towards a butyrogenic milieu (more adult like) may be of critical importance for a healthy maturation of the gut and the gut microbiota. Wopereis et al. (2017) proposed that this maturation process is associated with a reduced risk of developing eczema in infants at risk of developing allergies [64]. Moreover, butyrate has been shown to have regulatory effects on host immunity including anti-inflammatory mechanisms and has been generally associated with numerous health benefits by improvement of gut barrier function and pathogen inhibition [67].

## 4. Infant Type Bifidobacteria as Indigenous Probiotics 

Probiotics are live beneficial microorganisms which, when administered in adequate amounts, confer health benefits [68,69]. The conferred health benefits are mostly attributed to immunomodulation, restriction of pathogenic bacteria through competitive exclusion, SCFA production and modulation of mucosal barrier function [70]. Among early life microbial colonizers, members of genera *Streptococcus*, *Enterococcus*, *Bacillus*, *Escherichia*, *Propionibacterium* and *Lactococcus* and also yeasts such as *Saccharomyces* species, have been widely used as probiotics for a broad range of health benefits. Notably, bifidobacteria and lactobacilli are two of the most exploited taxonomic groups for probiotic applications [68,69]. Nonetheless, there have been documented efforts to characterize combinations of other commensal microorganisms directly isolated from healthy infants, for maintaining or even restoring normal gut microbiome composition to benefit host health [71]. 

Historically, the selection of probiotic strains for human applications is often based on technological criteria rather than ecological or clinical criteria [72]. In order to survive gastric passage and efficiently colonize and proliferate in the human gut, a probiotic strain needs to be tolerant to low pH, bile salts and proteolytic enzymes [68,69]. For some probiotics, adhesion to the intestinal mucosa is essential for colonization [73]. On the other hand, technological application requires resistance to processing conditions (including exposure to oxygen) and viability and stability in products over longer periods. Maintaining viability in products over a period of time (i.e., shelf-life) is often a major selection criterion for the choice of probiotics for commercial exploitation. Consequently, on technological grounds, *Lactobacillus* spp. are historically much more exploited for probiotic applications than other taxa such as *Bifidobacterium* spp. However, *Lactobacillus* spp., are sometimes found to be less adaptive to host conditions and may not sustain in the highly competitive ecological niche of the early life gut [74]. On the other hand, physiological properties of probiotics such as folate production, carbohydrate metabolic affinities and tolerance to stress are host dependent [32]. Therefore, eco-physiological adaptability and clinically relevant host–microbe interactions should be clearly very important parameters for the selection and development of probiotics. 

**Figure 2:**
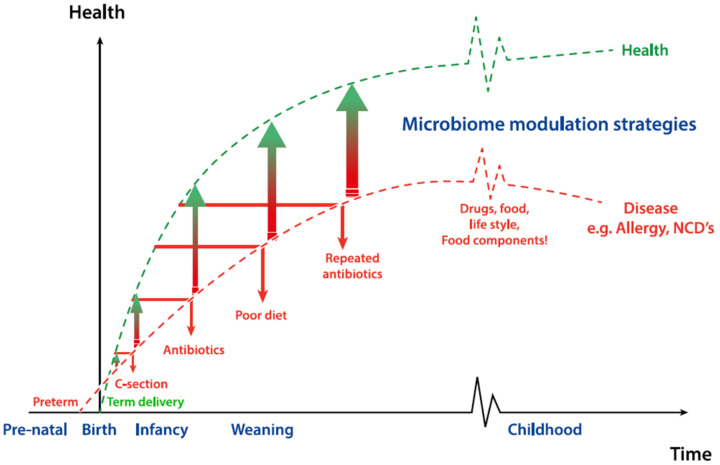
Impact of microbiome (including *Bifidobacterium* spp.) modulation strategies on early life and later life health outcomes. Advertent early life events such as pre-term birth or antibiotic exposure may already initiate a derailed microbial succession, which may be further amplified by poor diet and life-style, repeated antibiotic exposure, medications or disease. All these factors have been reported to contribute to the later life health outcomes such as allergy or non-communicable diseases. Restoration of the compromised microbiota involves accelerating the process of succession or attempting to change the trajectory of succession by microbiome modulation strategies.

Given the abundance in the early life gut, their HMO-driven symbiotic relationship with humans and the beneficial associations with health, bifidobacteria are often considered as ideal probiotics for infants. Delayed bifidobacterial colonization has been reported in cases of compromised delivery such as c-section and pre-term birth, or in the case of early life antibiotic exposure (Figure 2) [75]. Interestingly, bifidobacterial transmission is influenced by maternal factors before delivery. Nuriel-Ohayon et al. (2019) reported that there is a dramatic change in gut microbiota during pregnancy and bifidobacteria are enriched in late pregnancy—i.e., third trimester [76]. The authors also postulated that a rise in the level of *Bifidobacterium* is not only beneficial for healthy pregnancy but also reveals an evolutionary process that facilitates optimal transmission during birth and lactation [76,77]. In an independent study, it has been shown that women with excessive weight gain during pregnancy harbored lower numbers of *Bifidobacterium* spp. and *Bacteroides* spp. in their gut compared to pregnant women who had normal weight gain [78]. Therefore, bifidobacterial probiotics hold great potential for the restoration of compromised microbiota development, particularly in early life and are already used in currently commercialized probiotic infant nutrition products

### 4.1. Comparison with Probiotic Lactobacillus Species

*Lactobacillus* spp. have been extensively studied for probiotic applications and their general impact on host health. However, from an ecological perspective, only a small number of *Lactobacillus* spp. can be considered truly indigenous inhabitants of the human intestinal tract. Most industrialized *Lactobacillus* spp. have originally been isolated from fermented foods, the human oral cavity or sometimes proximal parts of the gastrointestinal tract [74]. Various studies have shown that the distribution of other dominant gut microbiota taxa, such as *Bifidobacterium*, *Bacteroides* and *Clostridia*, exhibit higher temporal stability compared to *Lactobacillus* spp. [74]. Especially in early life, *Lactobacillus spp.* are less abundant than *Bifidobacterium spp*. and their presence is mostly transient and driven by “chance colonization” processes immediately after birth [72]. Lactobacilli are members of the Phylum Firmicutes which are taxonomically and genetically distinct from the Phylum Actinobacteria to which the genus *Bifidobacterium* belongs. 

In particular, infant-type *Bifidobacterium* spp. have evolved with a genetic makeup which is adapted for metabolizing host derived glycans, while *Lactobacillus* spp. typically have a more diverse carbohydrate metabolizing capacity [79]. Based on their evolutionary ecology and carbohydrate fermentation capacity, members of the genus *Bifidobacterium* are more likely to be stable colonizers of the infant gut [79] which is a desirable attribute for probiotic applications. Probiotic attributes of infant type *Bifidobacterium* spp. and subspp. such as *B. bifidum*, *B. longum* subsp. *longum*, *B. longum* subsp. *infantis* and their species specific relevance in infant health have been extensively reviewed [80,81,82,83]. In the following section, we summarize scientific evidence available for *B. breve*-based probiotic solutions in early life development and specific clinical conditions. 

### 4.2. Beneficial Effects of Bifidobacterium Breve Strains on Infant Health

The history of dairy products supplemented with *Bifidobacterium* dates back to the early 1970s, and products have been marketed since the early 1980s in Japan [84]. Moreover, due to their eco-physiological function and perceived health benefits, *Bifidobacterium* spp. have been widely used as probiotic supplements for infants and young children. Specifically, probiotic applications of *Bifidobacterium breve* strains such as BBG-001, BR-03, B632, M-16V, BB536, CNCM I-4035 and C-50, have been documented [85]. 

*B. breve* strains have demonstrated antipathogenic, anti-inflammatory and immune-modulating properties [86]. Immune benefits of *B. breve* strains have been reported in infants with allergic disorders, in very low birth weight infants and in prevention of late-onset sepsis and necrotizing enterocolitis in preterm infants [87,88]. Apart from immune mediated health benefits, positive effects of *B. breve* strains have also been observed in children with antibiotic associated diarrhea [89], in pediatric patients undergoing chemotherapy [90] and in children with celiac disease [91]. 

*B. breve* strains combined with digestible substrates—i.e., prebiotics—may further the synergistic health effects on the host (Figure 2). The combination of scGOS/lcFOS and *B. breve* M-16V supplemented with an extensively hydrolyzed formula demonstrated to be well tolerated in healthy term infants, and supported an adequate infant growth [92]. In infants with suspected non-IgE mediated cow’s milk allergy, an amino-acid-based formula (AAF) supplemented with a synbiotic blend of fructo-oligosaccharides and *B. breve* M-16V was shown to improve the gut microbiota composition by modulating bifidobacterial levels and *Eubacterium rectale*/*Blautia coccoides* taxon levels closer to that of healthy breast fed infants [93]. 

*B. breve* strains also produce metabolites which can have a direct or indirect impact on the host health. For example, in a preclinical model, it was demonstrated that cell surface associated exopolysaccharide (EPS) of *B. breve* UCC2003 reduced the colonization of the gut pathogen *Citrobacter rodentium* [94]. Bacterial metabolites or bioactive compounds produced during the fermentation process—i.e., postbiotics—could also exert a beneficial effect on the host [95,96]. 

Postbiotics may include metabolites such as SCFA, saccharides such as polysaccharide A, secreted molecules such as lactocepin and p40 molecules [96]. Infant formula with postbiotics originating from fermentation by *S. thermophilus* (ST065) and *B. breve* C50 (BbC50) strains have been shown to enhance the production of intestinal sIgA [97] and resulted in less severe diarrheal episodes [98].

Another evolving strategy of gut microbiota modulation could include a combination of prebiotics and postbiotics. For instance, a clinical study with a partially fermented (postbiotic) formula supplemented with scGOS/lcFOS in a ratio of 9:1 resulted in reduced incidence of infantile colic and increased sIgA in addition to more bifidobacteria and less pathogenic bacteria such as clostridia-related species [99,100]. Taken together, these examples of microbiota modulation strategies based on probiotics/prebiotics/postbiotics or combinations thereof, are closely associated with the unique metabolic characteristics of *B. breve* strains.

### 4.3. Preterm Infants

Preterm birth (PTB), defined as birth at fewer than 37 weeks gestational age, is a major cause of neonatal morbidity and mortality. Globally, 14.8 million babies are born prematurely. In both developed and underdeveloped countries, PTB rates have not significantly decreased in the past 40 years and in some cases have increased [101].

Some of the common complications of preterm birth include high rate of respiratory distress syndrome, Necrotizing Enterocolitis (NEC), early- and late-onset sepsis, cerebral palsy, infections and feeding difficulties [101]. These health complications are mainly associated with immature organ systems that are not yet prepared to support life in the extrauterine environment. As a consequence of preterm delivery, the development of gut microbiota is also impacted and preterm infants have taxonomically less diverse microbiota with increased abundance of facultative anaerobes (Figure 2) [102,103,104]. These differences in microbiota composition could be attributed to the intensive care environment and extensive use of antibiotics after birth. This is supported by the observation that interindividual differences in microbiota composition of hospitalized very low birth weight infants becomes smaller with increasing stay [105,106]. More specifically, the microbiota of hospitalized infants converges toward a microbiota enriched with bacterial families *Enterobacteriaceae* and *Enterococcaceae*, including members of the genera *Klebsiella*, *Enterobacter* and *Clostridium* and depleted of beneficial genera such as *Lactobacillus* and *Bifidobacterium* [14,107]. 

Compromised microbiota composition in combination with under-developed immune system may leave preterm infants susceptible to contracting nosocomial infections, such as NEC and sepsis. Prophylactic treatment with broad-spectrum antibiotics, such as amoxicillin, ceftazidime, erythromycin and vancomycin, is common practice in neonatal wards for the prevention and treatment of infections and sepsis.

While antibiotics decrease mortality and morbidity rates on the one hand, they also pervasively disrupt early-life microbiota development and specifically delay *Bifidobacterium* colonization [108]. To mitigate the health risks associated with compromised microbiota development, probiotic supplementation is now increasingly recognized as routine therapy for preterm infants. *B. breve* M-16V is among the widely used probiotics in preterm infants and has been shown to significantly reduce the incidence of NEC [86,109]. Furthermore, supplementation with *B. breve* M-16V to extremely preterm infants was found to be effective in restoring the normal gut microbiota composition [109]. Furthermore, a systematic review of the applications of *B. breve* M-16V in preterm neonates concluded that *B. breve* M-16V is safe and showed no adverse effects. However, a multicenter clinical study that included 1300 preterm infants did not conclusively demonstrate a clinical benefit of supplementation with *B. breve*-001 [110], which corroborates that probiotic effects can be highly strain specific. Based on these mixed observations, adequately powered Randomized Clinical Trials (RCTs) are needed to confirm the findings and to support the routine use of bifidobacterial probiotics in preterm infants [111].

### 4.4. Cesarean Section

Globally, birth through c-section has almost doubled from year 2000 (12.1%) to year 2015 (21.1%) [112]. This extra-ordinary rise in c-section delivery may have health consequences. In particular, C-section is associated with an increased risk of immune diseases, such as asthma, eczema and allergies [113]. Although the mechanistic links between c-section and immune function are not fully established [114,115], there are ample reports that demonstrate the role of birth mode on early life microbiome colonization. It has been suggested that observed C-section effects on gut microbial colonization are associated with resulting unintended intrapartum exposure to broad-spectrum antibiotics in all C-section born neonates (Figure 2) [116]. However, Reyman et al. (2019) demonstrated by postponing antibiotic administration to mothers until after cord clamping that the effect of C-section birth on gut microbiota is largely independent of intrapartum antibiotics [117,118]. 

To restore microbiota development in c-section-delivered infants, there are multiple strategies which are being explored. One such strategy is vaginal seeding: inoculating a gauze or swab with vaginal fluids to transfer the vaginal flora to the mouth, nose or skin of a newborn infant. Evidence from a small pilot study indicated that vaginal seeding partially restores the microbiome of c-section delivered neonates [119], but larger, more rigorous studies are needed to assess the effect of vaginal seeding on microbiome trajectories and health outcomes. Of additional concern is that 20% of pregnant women at term are carriers of group B streptococci. Furthermore, undiagnosed carriage of *C trachomatis*, *N gonorrhea*, human papilloma virus and herpes simplex virus infections, among others, could result in adverse exposure to these pathogens. [120]. 

A more controlled strategy could be to use probiotics or prebiotics: for instance, *Lactobacillus rhamnosus* GG supplementation during pregnancy was found to be effective in modulating the gut microbiota and also resulted in enrichment bifidobacteria in neonates [121]. 

Barret et al. (2015) showed that prebiotic (GOS and poly-fructose) supplementation during the first four weeks of life led to increased prevalence of *Bifidobacterium longum* and also promoted bifidobacteria strain diversity [122]. 

A more recent clinical study by Chua et al. (2017) demonstrated that specific synbiotics—short chain galactooligosaccharides and long chain fructooligosaccharides (scGOS/lcFOS) in combination with *B. breve* M-16V—compensates the delayed *Bifidobacterium* colonization in C-section-delivered infants and modulates the production of acetate and the acidification of the gut. [123]. All these nutritional approaches are targeted to restore the complete microbiota composition and the gut milieu in c-section delivered neonates, which further substantiates the key role of vertical transfer and maintenance of *Bifidobacterium* spp. in neonates (Figure 2). 

## 5. Allergy Development

In the last 50 years, the global prevalence of allergic diseases has consistently increased and is expected to reach up to 4 billion people by 2050 [124]. Although there is a strong genetic link attributed to the perceived allergy epidemic, it rarely starts at birth. 

According to recent reports, early exposure to specific microbial taxa is quintessential for immune training. Suboptimal transfer of microbes such as in the case of infants born by c-section, exposed to antibiotics or formula feeding are risk factors attributed to allergy development (Figure 2). In a landmark study by Kalliomaki et al., it was shown that atopic infants at 3 weeks of age had significantly higher clostridia to bifidobacteria ratios [125]. This observation was further substantiated by another study conducted in Turkey, which showed that *B. longum* was present in significantly lower amounts in allergic children (age: 0–3 years) than in healthy controls [126]. A high level of adult-type bifidobacteria such as *B. pseudocatenulatum* and *B. catenulatum* and low level of infant type bifidobacteria such as *B. breve* were also found to be associated with eczema development [126,127,128]. These findings further support the relevance of bifidobacterial colonization in early life and suggest that inadequate transfer of bifidobacteria might precede allergy development. 

In children with atopic dermatitis (AD), it was demonstrated that the synbiotic supplementation (*B. breve M-16V* and scGOS/lcFOS) could prevent asthma-like symptoms, while in asthmatic adults, this synbiotic mix reduced allergen-induced immune responses [87,129]. Furthermore, in infants with AD, the combination of *B. breve* M-16V with scGOS/lcFOS did not show any effect on AD, while in a subgroup of infants with IgE associated AD, resulted in a significant reduction in AD [130]. In the case of infants with a cow’s milk allergy, amino acid formula when supplemented with *B. breve M-16V* and scFOS/lcFOS modulated the microbiome composition closer to the healthy breast-fed infants [93]. These findings support the hypothesis that inadequate microbiome colonization is key in the manifestation of allergic diseases and that Bifidobacterial levels in early life align with key stages in immune maturation. Studies have shown that bifidobacteria mediate a dialogue with mucosa-associated immune cells, having both pro- and anti-inflammatory effects promoting antipathogen immune responses [86,131]. 

Several studies show immune receptor–ligand interactions and immune signaling pathways linked to specific bifidobacterial compounds, such as pili and exopolysaccharide (EPS) [94,131,132,133]. Although, in most cases, the molecular mechanisms involved are not fully understood, these observations hold great promise for translation into microbiota modulation strategies for allergy prevention and management. 

## 6. Emerging Relevance of Bifidobacteria in Later Life

Although bifidobacterial predominance is most pronounced in infants, especially during lactation, it is still among the most abundant genera in adults [40]. Among all the bifidobacterial species, *B. adolescentis* is the most frequently isolated species in adults [134,135,136]. Genotypic and phenotypic characterization of *B. adolescentis* strains have revealed their extensive metabolic capabilities in utilizing diet derived glycans, such as starch, poly- and oligo-saccharides, amylopectin, pullulan, maltotriose and maltodextrin [137]. However, *B. adolescentis* lacks genes involved in metabolism of host-derived glycans such as mucin and human milk oligosaccharides, which differentiates it from other infant type bifidobacteria. 

In elderly people, there is a gradual decline in bifidobacterial abundance which is accompanied by decreased microbial diversity [40]. This has been repetitively confirmed by several studies using different technologies [138,139,140,141]. In a recent Japanese cross-sectional study, changes in *Bifidobacterium* abundance was investigated during the entire life span (age 0 – 104 years) (*n* = 441) [39]. The *B. longum* group was the most prevalent taxon across the life span, while *B. breve* was detected in almost 70% of children under the age of 3. In adults, total abundances of bifidobacterial species were low, but *B. adolescentis* and *B. longum* subsp. *longum* were found to be often prevalent in centenarians. *B. adolescentis* and *B. longum* subsp. *longum* have been hypothesized to benefit centenarians by enhancing immunity [39,142,143]. 

In old age, there is reduced immune tolerance, reduced immune memory and immune surveillance, and these immunological changes are associated with increased risk of infection and illnesses such as cystic fibrosis, hepatitis B and both diabetes Type 1 and 2 [40,144]. Specific probiotic strains are known for their immunomodulating properties; therefore, the application of probiotics is also gaining interest for applications in ageing populations. For instance, consumption of probiotic *Bifidobacterium lactis* Bi-07 in healthy elderly adults has been shown to enhance phagocytic activity of monocytes and granulocytes and thereby increased immune tolerance [145]. Elderly people commonly experience higher incidence of gastrointestinal symptoms such as constipation. A clinical study by Pitkala et al. (2007) demonstrated that probiotic administration in a fermented oat product—i.e., *B. longum* and *B. lactis*—led to an increase in the frequency of bowel movements in Finnish elderly subjects [146]. Taking into account all of these recent clinical observations, there is a clear potential for using bifidobacteria-based probiotics in adults, more specifically in the ageing population. 

## 7. Conclusions

Rapid changes in human lifestyles over the past 100 years, including profound changes in modern-day infant nutrition and birthing practices [147], have had a profound impact on early life microbiota acquisition [148] and may specifically impact the colonization by bifidobacteria. Immediately after birth, the physicochemical properties and the continued availability of HMOs offer a strong selective advantage for early-life type *Bifidobacterium* spp. Comparisons of infants with varying *Bifidobacterium* abundances in early life are necessary to understand how the loss of this keystone taxon and its critical ecological function impacts overall infant health and development.

Childbirth by c-section, premature birth and decreased limit of gestational viability, and exposure to intrapartum antibiotics during delivery, have become increasingly common in both developed and developing countries and have been very effective in saving maternal and infant lives. However, these deviations from the natural process of delivery also impact microbiome acquisition in infants and are increasingly being recognized as potential risk factors for diseases such as allergy. Increasing the bifidobacterial abundance by means of probiotics, prebiotics or postbiotics is among the evolving strategies to re-introduce bifidobacteria as a keystone species and hence impact health in early life. The *Bifidobacterium* sp. “types”, which are broadly categorized into two classes—infant type (i.e., dominant in early life) and adult type (i.e., dominant in adults)—further emphasize the importance of age appropriate probiotics. There is ample clinical evidence which supports the application of *B. breve* strains in children with allergy, born by c-section or born prematurely. More recently, bifidobacteria-based probiotics and synbiotics are also being investigated in adults and aging subjects and hence could contribute to overall health. Although probiotic applications are always strain specific, adequately designed clinical trials in larger cohorts of interest are still warranted. 

Microbiota transmission from mother to child is a controlled process. It has been demonstrated that the keystone microbes that drive early life gut microbiota development are acquired mostly vertically under ecological selection mechanisms, rather than through chance-driven processes. Nevertheless, a better understanding of the potential internal and external drivers of strain inheritance and selection by infants in early life is still needed.
